# Nicotine‐prevented learning and memory impairment in REM sleep‐deprived rat is modulated by DREAM protein in the hippocampus

**DOI:** 10.1002/brb3.704

**Published:** 2017-04-20

**Authors:** Norlinda Abd Rashid, Hermizi Hapidin, Hasmah Abdullah, Zalina Ismail, Idris Long

**Affiliations:** ^1^ BRAINetwork Centre for Neurocognitive SciencesSchool of Health SciencesUniversity Sains MalaysiaKubang KerianKelantanMalaysia; ^2^ School of Health SciencesUniversity Sains MalaysiaKubang KerianKelantanMalaysia

**Keywords:** DREAM protein, hippocampus, learning and memory, Morris water maze, nicotine, REM sleep deprivation

## Abstract

**Introduction:**

REM sleep deprivation is associated with impairment in learning and memory, and nicotine treatment has been shown to attenuate this effect. Recent studies have demonstrated the importance of DREAM protein in learning and memory processes. This study investigates the association of DREAM protein in REM sleep‐deprived rats hippocampus upon nicotine treatment.

**Methods:**

Male Sprague Dawley rats were subjected to normal condition, REM sleep deprivation and control wide platform condition for 72 hr. During this procedure, saline or nicotine (1 mg/kg) was given subcutaneously twice a day. Then, Morris water maze (MWM) test was used to assess learning and memory performance of the rats. The rats were sacrificed and the brain was harvested for immunohistochemistry and Western blot analysis.

**Results:**

MWM test found that REM sleep deprivation significantly impaired learning and memory performance without defect in locomotor function associated with a significant increase in hippocampus DREAM protein expression in CA1, CA2, CA3, and DG regions and the mean relative level of DREAM protein compared to other experimental groups. Treatment with acute nicotine significantly prevented these effects and decreased expression of DREAM protein in all the hippocampus regions but only slightly reduce the mean relative level of DREAM protein.

**Conclusion:**

This study suggests that changes in DREAM protein expression in CA1, CA2, CA3, and DG regions of rat's hippocampus and mean relative level of DREAM protein may involve in the mechanism of nicotine treatment‐prevented REM sleep deprivation‐induced learning and memory impairment in rats.

## 1 Introduction

Sleep is a natural physiological process in all living organisms, featured by two main stages: nonrapid eye movement (non‐REM) followed by a much shorter period of rapid eye movement (REM) sleep (Stickgold, 1998). Previous reports revealed that sleep contributes significantly to the process of memory and neural plasticity (McDermott et al., 2003; Peigneux, Laureys, Delbeuck, & Maquet, 2001; Samkoff & Jacques, 1991). Formation of memory in the brain consists of at least three stages: encoding, consolidation, and retrieval (Abel & Lattal, 2001). Sleep is particularly beneficial to the consolidation stage of memory storage. Manipulation of sleep during this stage will affect the consolidation of memory. It is also known that adequate sleep is essential for fostering connections among neuronal networks for memory consolidation in the hippocampus (Kim, Mahmoud, & Grover, 2005; McDermott, Hardy, Bazan, & Magee, 2006). In fact, hippocampal activity is increased during sleep after a learning task (Gais & Born, 2004; Gais et al., 2007).

It has been shown that sleep deprivation especially REM sleep causes memory deficit (Alhaider, Aleisa, Tran, & Alkadhi, 2011), reduced level of brain‐derived neurotrophic factor (BDNF) in the rat hippocampus (Guzman‐Marin et al., 2006), and dysregulation of cAMP‐response element binding protein (CREB) pathway (Havekes, Meerlo, & Abel, 2015). REM sleep deprivation impairs learning and memory processes by affecting hippocampal synaptic plasticity which is important for learning acquisition and memory consolidation (McDermott et al., 2003). REM sleep deprivation also has been shown to disrupt the role of N‐methyl‐D‐aspartate (NMDA) receptor in the dentate gyrus (DG) of the hippocampus which is necessary for normal synaptic plasticity in the hippocampus during learning and memory consolidation (Yang et al., 2008). Furthermore, REM sleep deprivation has been demonstrated to decrease long‐term potentiation (LTP) (Ishikawa et al., 2006) but increases long‐term depression (LTD) (Tadavarty, Rajput, Wong, Kumar, & Sastry, 2001) of neuron activity in CA1 and DG regions of the hippocampus.

Downstream regulatory antagonistic modulator (DREAM) protein has been found to involve in learning and memory processes (Fontan‐Lozano et al., 2009). Study has been shown that DREAM protein acts as a negative regulator of the key memory factor CREB protein in a Ca^2+^‐dependent manner (Naranjo & Mellstrom, 2012). Knockout of the DREAM gene can facilitate CREB‐dependent transcription and markedly enhances learning and synaptic plasticity with improved cognition (Fontan‐Lozano et al., 2009). DREAM protein also has been found to act as a negative regulator to the NMDA receptor function such as in hippocampal synaptic plasticity (Yin et al., 2010). Therefore, modulation of DREAM protein in hippocampal regions is suggested to be involved in the mechanisms of REM sleep deprivation‐induced learning and memory impairment in rats.

Acute nicotine treatment has been reported to prevent impairment of learning and memory (Aleisa, Alzoubi, & Alkadhi, 2011; Aleisa, Helal, et al., 2011) and also LTP of the hippocampal CA1 region due to chronic psychosocial stress (Aleisa, Alzoubi, Gerges, & Alkadhi, 2006). In addition, acute nicotine treatment also has been shown to prevent enhancement of LTD in hippocampal CA1 region due to chronic stress (Aleisa, Alzoubi, & Alkadhi, 2006). However, the underlying mechanism of nicotine‐prevented learning and memory impairment due to REM sleep deprivation is still elusive. Therefore, this study was conducted to investigate whether the changes in hippocampal DREAM protein expression could be associated with acute nicotine treatment‐prevented learning and memory impairment in REM sleep‐deprived rats.

## 2 Materials and Methods

### 2.1 Animal preparation

Seventy‐two adult male Sprague Dawley (230–280 g) rats were obtained from the Animal Research and Service Centre (ARASC), Universiti Sains Malaysia, Kubang Kerian, Kelantan, Malaysia, were placed in a room with 12‐hr light/dark (lights on at 7:00 am and turn off at 7.00 pm) cycle at 28OC, with free access to food and water. There were six groups of rats in this study: control (C, *n *= 12), control treated with nicotine (C + N, *n *= 12), wide platform (W, *n *= 12), wide platform treated with nicotine (W + N, *n *= 12), REM sleep deprivation (REMsd, *n *= 12), and REM sleep deprivation treated with nicotine (REMsd + N, *n *= 12). The nicotine groups (C + N, W + N, and REMsd  +  N) were treated with 1 mg/kg nicotine (Sigma, St. Louis, MO, USA) subcutaneously twice a day, for 72 hr. The nontreatment groups were treated with subcutaneous saline injection. The nicotine dose in this study was based on previous study which was known to produce nicotine blood levels similar to those of chronic smokers (Le Houezec, Jacob, & Benowitz, 1993). All experiments were approved by the Animal Care and Use Committee of Universiti Sains Malaysia [USM/Animal Ethics Approval/2012/ (81) (408)].

### 2.2 Induction of REM sleep deprivation

The inverted flowerpot method was modified based on our previously study (Siran, Ahmad, Abdul Aziz, & Ismail, 2014) and used to selectively induce REM sleep deprivation for 72 hr. Before the start of the experiment, all rats except from control group were isolated and adapted individually in a dry tank model for 72 hr before being exposed to the REM sleep deprivation model. The control group rats were adapted in their normal dry cage for 72 hr. The purpose of the adaptation was to expose and adapt the rat to the glass tank environment before being exposed to the REM sleep deprivation model. For REM sleep deprivation procedure, two small platforms of 6.5 cm diameter, 8.5 cm height, and 8 cm length between both platforms were placed in a glass tank measuring 50 cm in height, 50 cm in width, and 100 cm in length. The rats were deprived of REM sleep by placing one rat at a time on top of one of the two platforms (6.5 cm in diameter) placed in the middle of a glass tank filled with water (platform was 1 cm above water) for 72 hr. REM sleep was prevented by the muscular atonia due to the rats awakening when the body came into contact with water. For wide platform group, each rat was placed in the same experimental condition as REM sleep deprivation model except that the diameter of the platform was larger (14 cm diameter, 8.5 cm height, and 8 cm length between both platforms) which allowed REM sleep to occur. The purpose of this W group was to expose them to the same aquatic environment as the REM sleep deprivation rat but allowed them to experience both NREM and REM sleeps ad libitum (May et al., 2005). This platform technique has been validated by other studies using electroencephalography and has been shown to deprive a small degree of non‐REM sleep (Maloney, Mainville, & Jones, 1999). The temperature of the water in the tub was 30°C. Food and water were available ad libitum throughout the time the rats were on the platforms.

### 2.3 Morris Water Maze test

The water maze test is a widely accepted method for learning and memory test, and this test was performed as described by (Vorhees & Williams, 2006). Maze testing was performed by the SMART‐CS (Panlab, Barcelona, Spain) program and equipment. A circular plastic pool (110 cm in diameter and 20 cm in depth) was filled to a depth of 14 cm with milky water kept at 22–25°C. An escape platform was submerged 0.5–1.0 cm below the surface of the water in position. On training trials, the rats were placed in a pool of water, and allowed to remain on the platform for 10 s and were then returned to the home cage during the second‐trial interval. The rats that do not find the platform within 60 s were placed on the platform for 10 s at the end of trial. They were allowed to swim until they sought the escape platform. These trials were performed in single platform and in three rotational starting positions. Escape latency, swimming distance, and swimming speed of each rat were monitored by a camera above the center of the pool connected to a SMART‐CS program (Panlab).

A probe trial in order to assess memory consolidation was performed 24 hr after the 5‐day acquisition tests. In this trial, the platform was removed from the tank, and the rats were allowed to swim freely. For these tests, the percentage time in the target quadrant and target site crossings within 60 s was recorded. The time spent in the target quadrant was recorded to indicate the degree of memory consolidation that has taken place after learning. The time spent in the target quadrant was used as a measure of spatial memory. Swimming pattern of each rat was monitored by a camera above the center of the pool connected to a SMART‐CS program described above.

### 2.4 Immunohistochemistry analysis

Rats were sacrificed by an overdose intraperitoneal injection of sodium pentobarbitone. This method was used to avoid damage to the spinal cord (Hao, Takahata, Mamiya, & Iwasaki, 2002). Thoracotomy was done to expose the heart. An 18G branula was then inserted into the left ventricle, and a snip was made to the right atrium for outlet. Perfusion was performed using gravity method with phosphate‐buffered saline (PBS) followed by 500 ml of cold 4% paraformaldehyde in 0.1 mol/L phosphate buffer (PB) (pH 7.4) (Hayati et al., 2008). The brain was dissected out from the rats. Following overnight cryoprotection in 20% sucrose in 0.1 mol/L PB, the brain was cut using cryostat and the hippocampus region was collected as free‐floating section in PBS. Sections then were rinsed with Tris‐buffered saline (TBS) and incubated overnight with rabbit polyclonal primary antibodies (dilution 1:500) for DREAM protein (Pierce, USA). Then, the sections were incubated with biotinylated secondary antibody anti‐rabbit (dilution 1:200) for 1 hr. After 1 hr, sections were reacted with avidin–biotin complex (ABC) and stained with diaminobenzidine and hydrogen peroxide until brown coloration was seen. Sections were mounted on slides, air‐dried, dehydrated, and covered with a cover slip.

Six tissue sections were randomly taken from one rat each group; therefore, the total tissue sections that were taken from one group were 36 tissue sections (*n *= 6). The tissue sections were scrutinized at hippocampal CA1, CA2, and CA3 and dentate gyrus regions. The DREAM positive neuron in every unit area (360,000 μm^2^) was then identified and captured at a 40× magnification. On average, four area units, that is, three in the hippocampus and one in the dentate gyrus, could be obtained from each section. The mean number of DREAM positive neuron per unit area in the hippocampus and dentate gyrus of each animal group was then calculated.

### 2.5 Western Blot analysis

In order to verify the DREAM protein quantification results, we performed Western blot analysis on the hippocampal protein extract. The brain tissue was removed directly from the rats without a fixation process being performed and separated into hippocampus region. The hippocampus region was immediately deep freezer by liquid nitrogen and kept at −80°C until further analysis. Protein was extracted from the hippocampus region using the NE‐PER extraction reagents (Pierce). Before being used, NE‐PER extraction reagents were mixed with concentrated Halt^™^ Protease Inhibitor cocktail kit, EDTA‐free (Pierce) in a volume of 10 μl/ml per reagent. The protein concentration of extracted samples was measured using the bicinchoninic acid (BCA) protein assay kit. Sample protein containing 40–50 μg total protein (after optimization) was denatured and subjected to SDS‐PAGE using 12% resolving gel. The protein from polyacrylamide gels was transferred to nitrocellulose membrane (Bio‐Rad, USA). The nitrocellulose membrane was washed with deionized water before incubated in blocking solution (5% BSA in PBS) for 1 hr at room temperature. Following that, the nitrocellulose membrane was washed three times for 10 min in Tris buffer saline‐Tween 20 (TBS‐T20). The nitrocellulose was then incubated with rabbit polyclonal DREAM antibody (dilution 1: 500 in TBST) or mouse monoclonal ß actin antibody (dilution 1:2000 in TBST,) overnight at 4°C. The nitrocellulose membrane was then incubated with HRP‐conjugated goat anti‐rabbit antibody (dilution 1:5000 in TBST) or mouse secondary antibody (dilution 1:5000 in TBST) for 1 hr at room temperature. In between incubations, the nitrocellulose membrane was washed three times in TBS‐T20 for 10 min each. Finally, the blot was examined using Immobilon Western Chemiluminescent HRP substrate and an image was taken using an image analyzer. The integrated density values (IDV) of DREAM and ß‐actin protein were measured using Spot Denso AlphaView^™^ software programmed in the image analyzer. The mean relative intensity or fold change was determined by the following formula:

$$\begin{array}{cc} \text{Mean Relative Intensity}= & (\text{IDV DREAM protein}\\ & -\text{IDV endogenous control)}\;\;\;\text{target group}\\ & -(\text{IDV DREAM protein}\\ & -\text{IDV endogenous control) calibrator group} \end{array}.$$

### 2.6 Statistical analysis

Statistical analyses were performed using Statistical Package of Social Sciences (SPSS) software version 21. Escape latency, swimming distance, and speed were analyzed using repeated measurements, and differences between specific groups were analyzed by analysis of variance (ANOVA) and Bonferroni post hoc test. The differences between specific groups for time spent in target quadrant, DREAM positive neuron expression and mean relative of DREAM protein level were measured using one‐way ANOVA, Bonferroni, and Dunnet's post hoc test. All data are reported as mean ± standard error mean (SEM), and the level of significance was set at *p *< .05.

## 3 Results

### 3.1 Escape latency and swim distance

**Figure 1:**
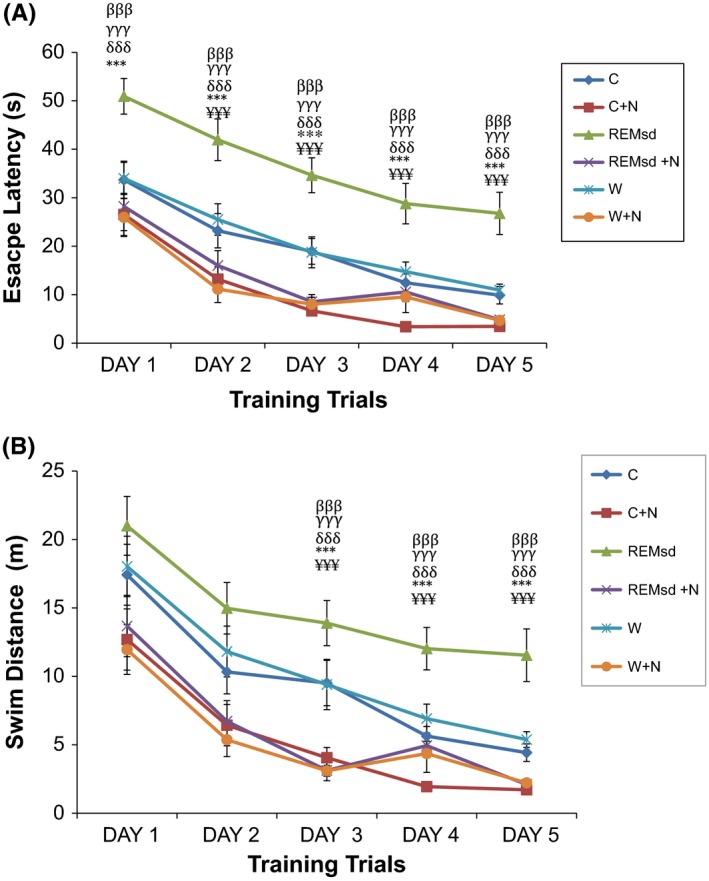
Escape latency time by Morris water maze test (A). Swim distance by Morris water maze test (B). The results are reported as mean ± SEM. ^¥¥¥^, *p *< .001 compared between REMsd and C, ***, *p *< .001 compared between REMsd and C + N, ^δδδ^, *p *< .001 compared between REMsd and R + N, ^γγγ^, *p *< .001 compared between REMsd and W, ^βββ^, *p *< .001 compared between REMsd and W + N

By using the Morris Water Maze test, we found that within training trial days, rats in all groups showed significant improvement in escape latency time to finding the submerged platform (*p *< .001) and swim in short distance (*p *< .001). However, when compared between groups, the REM sleep‐deprived group (REMsd) took the longest time to locate the hidden platform compared to the other groups (*p *< .001) in all training trial days. Similar pattern could be seen in swim distance, in which the REMsd group took the longest route before reaching the target platform compared to the other groups which swim in shorter distance and to escape on the platform (*p *< .001) (Figure 1). When comparison makes between nicotine‐treated and nontreated groups, all nicotine‐treated groups took least time and swim in shorter distance to find the submerged platform with the (C + N) group showed the shortest time and swim in short distance to find the target platform, while wide platform (W) and the control (C) groups took similar time and swim distance to get to submerged platform (Figure 1).

### 3.2 Probe test and Swim speed

**Figure 2:**
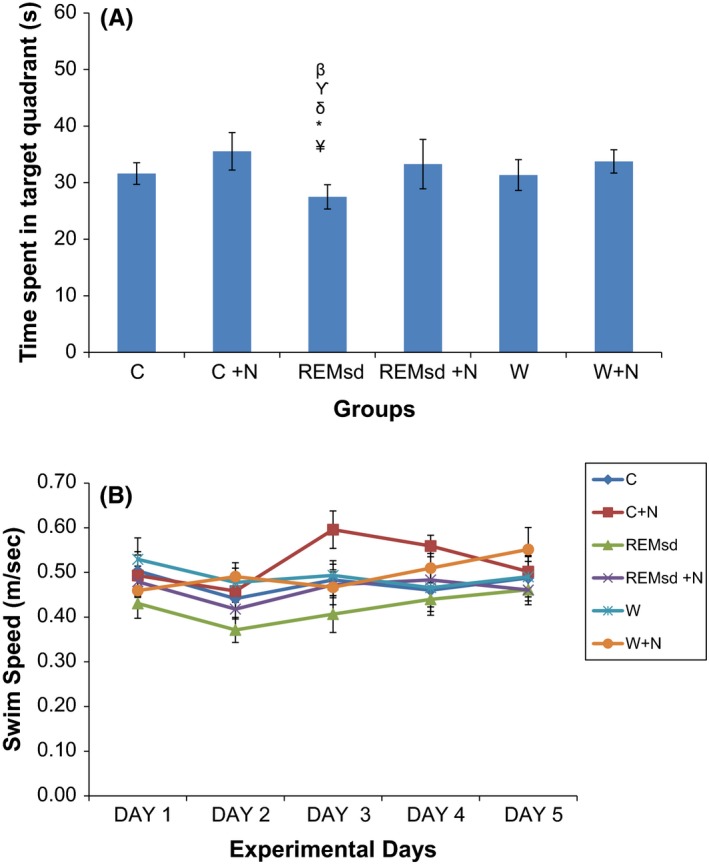
Time spent in target quadrat during probe test (A). Swim speed during Morris water maze test (B). The results are reported as mean ± SEM. ^¥^, *p *< .05 compared between REMsd and C, *, *p *< .05 compared between REMsd and C + N, ^δ^, *p *< .05 compared between REMsd and REMsd + N, ^γ^, *p *< .05 compared between REMsd and W, ^β^, *p *< .05 compared between REMsd and W + N

During probe test, experimental rats will try to remember the hidden platform location, and swim around the quadrant in which the platform was located previously. In general, all rats were swimming mostly in quadrant 1 as the hidden platform was located in this quadrant. We found that rats in REMsd group spent least time in quadrant 1 when compared to other groups during probe test (*p *< .05) (Figure 2A). However, there was no significant difference in swim speed within groups or comparison between all groups in all training trial days (Figure 2B).

### 3.3 DREAM protein expression in hippocampus

**Figure 3:**
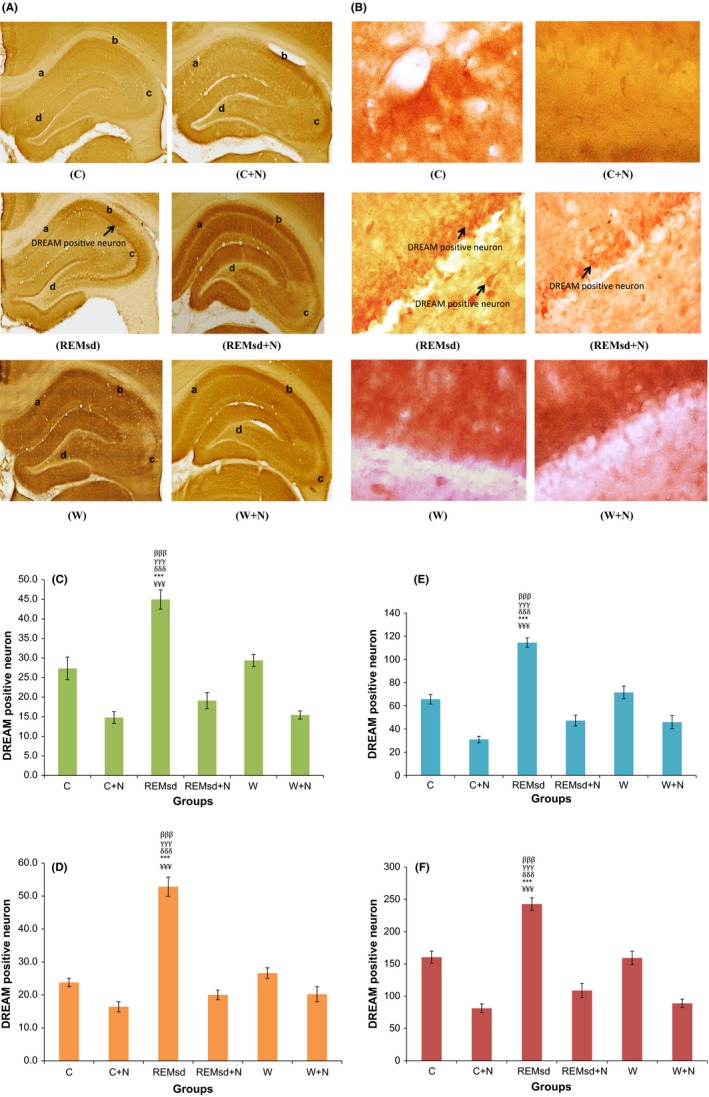
Immunohistochemistry results show the DREAM positive neuron (Arrow mark) (4× magnification) at CA1 (A), CA2 (B) CA3 (C) and DG (D) of hippocampus regions of all experimental groups (A), DREAM positive neuron (Arrow mark) (40× magnification) at DG region of all experimental groups (B). Mean DREAM positive neurons by immunohistochemistry analysis on CA1 (C), CA2 (D), CA3 (E), and DG (F) of hippocampus regions. The results are reported as mean ± SEM. ^¥¥¥^, *p *< .001 compared between REMsd and C, ***, *p *< .001 compared between REMsd and C + N, ^δδδ^, *p *< .001 compared between REMsd and REMsd + N, ^γγγ^, *p *< .001 compared between REMsd and W, ^βββ^, *p *< .001 compared between REMsd and W + N

DREAM protein expression was significantly increased in the REMsd group when compared to the other groups in CA1 (*p *< .001) (Figure 3C), CA2 (*p *< .001) (Figure 3D), CA3 (*p *< .001) (Figure 3E), and dentate gyrus hippocampus (*p *< .001) regions (Figure 3F). Treatment with nicotine significantly attenuated this effect and decreased DREAM protein expression in CA1, CA2, CA3 and dentate gyrus hippocampus regions as showed in REMsd + N group when compared to REMsd group (*p *< .001) (Figure 3C–F).

### 3.4 Mean relative of DREAM protein level in hippocampus

**Figure 4:**
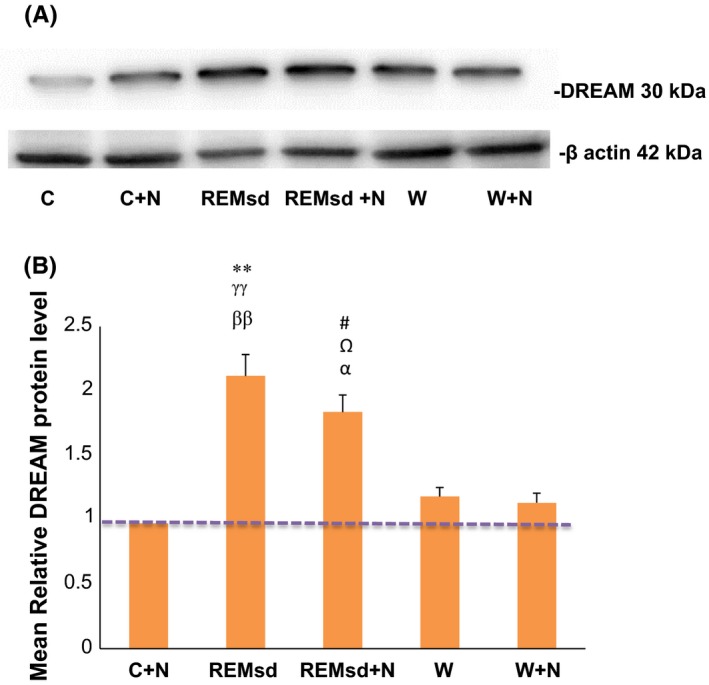
A representative example of Western blot results for DREAM protein level. The lower panel shows the loading control (A). Quantification analysis of the integrated density value (B). Columns represent the mean relative DREAM protein level ± SEM for six separate experiments. The mean relative DREAM protein level (fold change) represents comparative levels of the DREAM protein in the experimental group (F group) over the calibrator group (C group) after normalization by its loading control (housekeeping protein, β actin protein), *n *= 6 for each group. **, *p *< .01 compared between REMsd and C + N, ^γγ^, *p *< .01 compared between REMsd and W, ^ββ^, *p *< .01 compared between REMsd and W + N, ^#^, *p *< .05 compared between REMsd  + N and C + N, ^Ω^, *p *< .05 compared between REMsd + N and W, ^α^, *p *< .01 compared between REMsd  + N and W + N

Mean relative of DREAM protein level in hippocampus was significantly increased in the REMsd group when compared to the C + N (*p *< .01), W (*p *< .01), and W + N groups (*p *< .01) (Figure 4A,B). Treatment with nicotine (REMsd + N) slightly reduced this effect but not significantly decreased mean relative of DREAM protein level in hippocampus when compared to REMsd group. In addition, mean relative of DREAM protein level in hippocampus of the REMsd + N group was still higher if compared to the C + N (*p *< .05), W (*p *< .05), and W + N groups (*p *< .05) (Figure 4A,B).

## 4 Discussion

The aim of the present work was to investigate the association of DREAM protein in the underlying mechanism of nicotine treatment‐prevented learning and memory impairment due to REM sleep deprivation. The results from Morris water maze test agree with the previous studies which state that nicotine treatment can prevent impairment of learning and memory due to REM sleep deprivation (Aleisa, Alzoubi, & Alkadhi, 2011; Aleisa, Helal, et al., 2011). In this study, we found that REM sleep deprivation significantly affected learning and memory performance without affecting locomotor functions (swim speed) as demonstrated by the insignificant difference in swim speed of the REMsd groups with the all groups. Rats in REMsd groups took more time to learn finding hidden platform during training trial days, as well as wandering around (more travel distance) in order to escape from the water compared to the other groups. Treatment of nicotine significantly ameliorated the impairment of learning and memory as demonstrated in the nicotine treatment group especially in the REMsd + N group.

The underlying mechanism on how nicotine treatment can prevent learning and memory impairment due to REM sleep deprivation is still unclear. Studies have suggested that nicotine treatment can activate pre‐synaptic nicotine receptors that lead to the increase in glutamate release from the pre‐synaptic terminal and as a consequence increased the activity of excitatory neurons (Gray, Rajan, Radcliffe, Yakehiro, & Dani, 1996). Nicotine treatment could also facilitate the activity of excitatory neurons through desensitization of α7nAch in GABAergic neuron and reduce the release of GABA (Tang et al., 2011). In addition to that, chronic nicotine treatment has been demonstrated to reverse stress‐induced reductions in protein levels of the brain‐derived neutrophic factor (BDNF) (Aleisa, Alzoubi, & Alkadhi, 2011), a key protein in hippocampal synaptic plasticity (Lu, Christian, & Lu, 2008). Thus, preventing sleep deprivation‐induced impairment of memory using nicotine is an exciting finding.

In this study, we found that hippocampal DREAM protein expression and the mean relative of DREAM protein level significantly increase in REMsd groups compared to other groups. Upregulation of DREAM protein was significantly higher at hippocampal CA1, CA2, and CA3 and DG regions of REMsd group compared to other groups (Figure 3C–F). The mean relative of DREAM protein level in hippocampus also was significantly higher in REMsd groups compared to C + N, W, and W + N groups (Figure 4A,B). The higher DREAM protein expression in all hippocampal regions was abolished after nicotine treatment but only slightly reduce the mean relative of DREAM protein level as shown in the REMsd + N group. The decreased hippocampal DREAM protein expression in the REMsd + N groups was consistent with the reversible effects on the learning and memory impairment as shown by the Morris water maze test. These results suggest that the modulation of DREAM protein expression associates with the effects of nicotine treatment in preventing learning and memory impairment due to REM sleep deprivation. However, we found that the mean relative of DREAM protein level in hippocampus of the REMsd + N group was slightly reduced when compared to the REMsd groups. This result could be due to the way of analysis was done. Western blot analysis is semi‐quantitative measurement and this possibly could affect the results in this study.

However, until today, the association between DREAM protein and REM sleep deprivation in the mechanism of learning and memory is still uncertain. DREAM protein has been demonstrated to be involved in the mechanism of learning and memory by functioning as a transcriptional repressor for CREB in a calcium‐dependent manner. DREAM gene knockout mice have been reported to facilitate the CREB gene transcription and enhanced learning and memory performance (Fontan‐Lozano et al., 2009). A study using transgenic mice overexpressing a Ca^2+^‐insensitive DREAM mutant (TgDREAM) found that DREAM protein played a role in postsynaptic modulation of the NMDA receptor and contributed to synaptic plasticity and also behavioral memory (Wu et al., 2010). The mice lacking the DREAM protein were found to facilitate the learning and memory process by decreasing potassium A current (I_A_). The results were comparable when the mice were treated with 4‐aminopyridine (4‐AP, 1 mg/kg i.p) (I_A_ inhibitor). The decreased potassium A current (I_A_) has been shown to require the activation of NMDA receptors containing the NR2B subunit to facilitate the learning and memory process (Fontan‐Lozano et al., 2009). These situations can be created in the neuron of brain hippocampus due to REM sleep deprivation by stimulating the release of intracellular calcium that induced noradrenaline‐mediated increase in Na‐K‐ATPase activity in rat brain (Das, Gopalakrishnan, Faisal, & Mallick, 2008), disturbing excitatory and inhibitory neurotransmitter release in the hippocampus and electrophysiological changes in the brain that may affect brain proper functionality (Fontan‐Lozano, Suarez‐Pereira, Delgado‐Garcia, & Carrion, 2011; Mohammed, Aboul Ezz, Khadrawy, & Noor, 2011; Xie et al., 2015).

## 5 Conclusion

This study suggests that DREAM protein may involve in the mechanism of nicotine treatment‐prevented learning and memory impairment due to REM sleep deprivation by changes in its expression level in all hippocampus regions of REM sleep‐deprived rats. However, the exact mechanism on how DREAM protein plays the role in the hippocampal plasticity and synaptic transmission and how these proteins interact with other proteins that involves in learning and memory during nicotine treatment in REM sleep deprivation is still unclear. Therefore, further investigation is needed especially on the glutamic acid (GLU) and γ‐amino‐butyric acid (GABA) levels in the brain tissue, brain‐derived neurotrophic factor (BDNF), cyclic AMP response element binding protein (CREB), and phosphorylated‐CREB (p‐CREB) protein expressions in the nicotine‐treated REM sleep‐deprived rat hippocampus in order to elucidate the mechanism.

## Conflict of Interest

The authors have no disclosures to declare.
